# Testosterone Modifies Alterations to Detrusor Muscle after Partial Bladder Outlet Obstruction in Juvenile Mice

**DOI:** 10.3389/fped.2017.00132

**Published:** 2017-06-07

**Authors:** Andrew S. Flum, Paula R. Firmiss, Diana K. Bowen, Natalie Kukulka, Grace B. Delos Santos, Robert W. Dettman, Edward M. Gong

**Affiliations:** ^1^Department of Urology, Northwestern University Feinberg School of Medicine, Chicago, IL, United States; ^2^Developmental Biology, Stanley Manne Children’s Research Institute, Chicago, IL, United States; ^3^Department of Urology, Loyola University Health System, Maywood, IL, United States

**Keywords:** testosterone, fibrosis, bladder outlet obstruction, posterior urethral valves, lower urinary tract diseases, bladder fibrosis, androgen

## Abstract

Lower urinary tract symptoms secondary to posterior urethral valves (PUV) arise in boys during adolescence. The reasons for this have previously been attributed to increased urine output as boys experience increased growth. Additionally, there are few choices for clinicians to effectively treat these complications. We formed the new hypothesis that increased androgen levels at this time of childhood development could play a role at the cellular level in obstructed bladders. To test this hypothesis, we investigated the role of testosterone on bladder detrusor muscle following injury from partial bladder outlet obstruction (PO) in mice. A PO model was surgically created in juvenile male mice. A group of mice were castrated by bilateral orchiectomy at time of obstruction (CPO). Testosterone cypionate was administered to a group of castrated, obstructed mice (CPOT). Bladder function was assessed by voiding stain on paper (VSOP). Bladders were analyzed at 7 and 28 days by weight and histology. Detrusor collagen to smooth muscle ratio (Col/SM) was calculated using Masson’s trichrome stain. All obstructed groups had lower max voided volumes (MVV) than sham mice at 1 day. Hormonally intact mice (PO) continued to have lower MVV at 7 and 28 days while CPO mice improved to sham levels at both time points. In accordance, PO mice had higher bladder-to-body weight ratios than CPO and sham mice demonstrating greater bladder hypertrophy. Histologically, Col/SM was lower in sham and CPO mice. When testosterone was restored in CPOT mice, MVV remained low at 7 and 28 days compared to CPO and bladder-to-body weight ratios were also greater than CPO. Histologic changes were also seen in CPOT mice with higher Col/SM than sham and CPO mice. In conclusion, our findings support a role for testosterone in the fibrotic changes that occur after obstruction in male mice. This suggests that while other changes may occur in adolescent boys that cause complication in boys with PUV, the bladder itself responds to testosterone at the cellular level. This opens the door to a new understanding of pathways that influence bladder fibrosis and could lead to novel approaches to treat boys with PUV.

## Introduction

Posterior urethral valves (PUV) is the most common congenital cause of pediatric bladder outlet obstruction (1). While mortality rate from PUV has significantly improved with modern medical management, obstructive uropathy still remains a significant source of morbidity into adulthood (2). Up to 80% of children with PUV display symptoms of incontinence and urinary tract infections (3). Furthermore, up to 50% of children with PUV will develop kidney failure, with obstructive uropathy the most common congenital cause of renal transplantation (4). The obstructive valve leaflets are surgically treated soon after birth; however, children with PUV will develop variability in urinary bladder function throughout their lives. It has been demonstrated that bladder deterioration often occurs following puberty, with an increasing need for clean intermittent catheterization (5).

Studies utilizing various animal models have demonstrated the bladder to be hormonally sensitive. Specifically, testosterone replacement has been shown to increase smooth muscle mass and smooth muscle to collagen ratio in orchiectomized rats (6). A model of partial bladder outlet obstruction in male mice was studied and shown to produce bladder detrusor muscle hypertrophy (7). Testosterone has been shown to increase detrusor fibrosis in response to ischemia–reperfusion injury in castrated rabbits (8). However, to date, the effect of hormonal manipulations on the response of the male mouse bladder to injury produced by obstruction has yet to be studied. Therefore, we hypothesize that testosterone participates in the maladaptive fibrotic changes that occur following bladder outlet obstruction.

## Materials and Methods

### Murine Partial Bladder Outlet Obstruction Model

All surgical procedures were approved by the Animal Care and Use Committee of the Stanley Manne Children’s Research Institute. Eight- to ten-week CD1 male mice (Charles River Labs, Chicago, IL, USA) were housed in separate cages to avoid changes to testosterone levels caused by group housing. Mice were anesthetized with 3% isoflurane and positioned supine. The lower abdomen was shaved and prepared with providone-iodine solution. After ensuring satisfactory induction of anesthesia, a midline lower abdominal skin incision was made and carried down through the abdominal wall. Bladder outlet obstruction was carried out in a fashion similar to a previous report, but with some differences (partial obstruction or PO group) (7). Please note that partial obstruction is typically referred to as “pBOO,” however, we will refer to the surgery and experimental group as PO. With upward traction on the bladder, blunt dissection around the bladder neck (proximal to the prostate in male mice) was carried out while occasionally retracting the bladder caudally to ensure dissection to be anterior to the ureters. A 7-0 polypropylene suture was then passed around the bladder neck and tied down over a 23-gauge needle placed next to the urethra. The needle was then removed. If male animals were to be castrated (castrate partial obstruction or CPO), simultaneously the testes were brought out of the abdominal incision, the spermatic cords ligated with 6-0 polypropylene sutures and sharply divided. The abdominal wall and skin were then closed separately with 5-0 polyglactin sutures in an interrupted fashion. Sham animals underwent an identical procedure up to the point of bladder mobilization. Animals were allowed to recover, provided carprofen dissolved in drinking water, and sacrificed at 1 or 4 weeks following functional studies described below. Following sacrifice by cervical spine dislocation under isoflurane anesthesia, animals were weighed and bladders were dissected out at the level of the ligature. Each bladder was emptied of urine and then separately weighed for 12 sham, 17 PO, 11 CPO, and 11 CPOT mice.

### Testosterone Administration

Testosterone cypionate was administered to the castrate, partially obstructed group at time of castration and weekly postoperatively at dose of 10 mg/kg (castrate partial obstruction testosterone or CPOT group). For mice given testosterone replacement, blood was drawn at time of sacrifice and sent for commercial testosterone assay to ensure they were receiving adequate replacement (Antech Diagnostics, Irvine, CA, USA).

### Voiding Stain on Paper (VSOP)

VSOP was performed the mornings of post-operative days 1, 7, and 28 for each mouse. Mice were maintained in metabolic cages with underlying Whatman filter paper for 2 h and given unrestricted access to food and water. Filter paper sheets were imaged. Number and pattern of voids was noted and the area of each voiding stain was measured using ImageJ (National Institutes of Health, Bethesda, MD, USA). Using a standard curve created from voiding stains from known volumes of urine, stain area was converted to voided volume. Max voided volume was analyzed.

### Histological Analysis

Bladders were fixed in 10% (w/v) formalin (Sigma) for 24 h, embedded in paraffin and cut transversely with the bladder neck oriented down to produce 4 µm sections. Sections from 4 sham, 4 PO, 4 CPO, and 4 CPOT mice were stained with Masson’s trichrome. Sections were imaged at 10× and 40× magnification using a Leica upright microscope connected to a camera and images were digitally stored using OpenLab for Mac. Col/SM ratio was quantified using Adobe Photoshop as previously described and averaged from four 40× sections per bladder. For immunostaining, bladders were fixed in 4% (w/v) formaldehyde made from paraformaldehyde (EM Sciences) overnight. Tissue was washed in DPBS and equilibrated sequentially in 20% (w/v) and 30% (w/v) sucrose (Sigma) in DPBS. Tissues were frozen in peel-away molds containing OCT (Tissue Tech) in a dry ice isopentane (Sigma) bath.

### Immunofluorescence Antibodies

Fixed bladders from 3 sham, 3 PO, 3 CPO, and 3 CPOT mice were utilized. Sections of 10 µm from were cut for all experiments. Sections were washed twice in PBS, incubated for 5 min at room temperature in PBS + 0.1% Tween (PBT), and blocked for 30 min at room temperature with 10% donkey serum in PBT. Smooth muscle myosin (SMM) was detected the same way using rabbit anti-smooth muscle myosin (Biomedical Technologies, 1:100) in conjunction with the Alexa Fluor 546 donkey anti-rabbit secondary antibody (Life Technologies, 1:400).

### Statistical Analysis

Groups were compared using ANOVA with *post hoc* Student–Newman–Keuls analysis. Results with *P* < 0.05 were considered significant. SPSS v22.0 was utilized for statistical analysis. Error bars in figures reflect SEM.

## Results

### PO Leads to Functional Changes Determined by Urodynamic Studies and Voiding Stain on Paper

**Figure 1:**
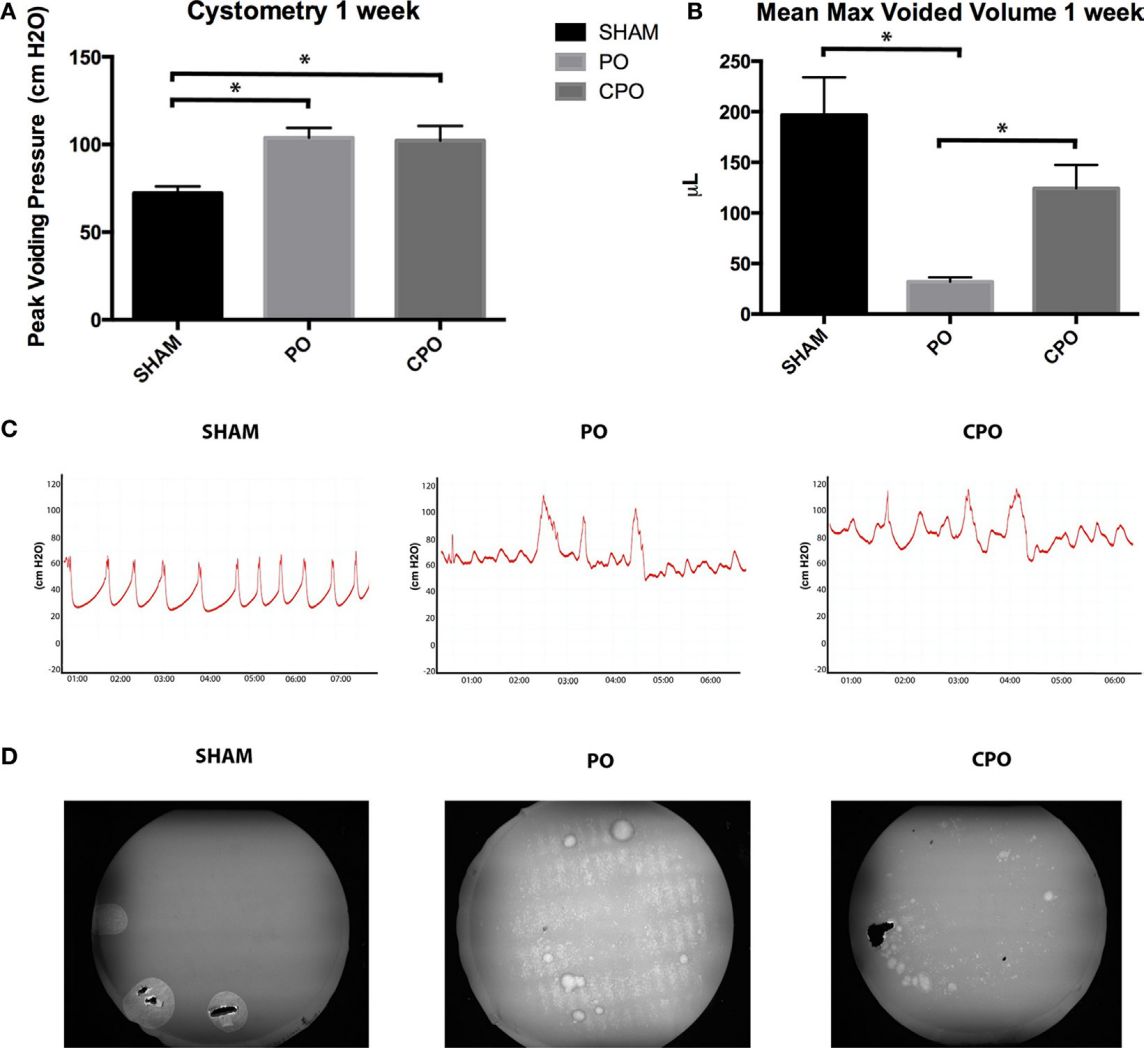
Cystometry **(A)** and mean maximum voided volumes **(B)** from mice 1 week after undergoing PO. Columns are (from left to right) Sham, PO, CPO. **(C)** Representative tracings from cystometry analysis. **(D)** Representative images of voiding stains on Whatman Paper placed under mice for 2 h as visualized with ultra violet imaging. Error bars are SEM. Statistical significance (*P* < 0.05) is indicated above bars with an asterisk.

Partial bladder outlet obstruction was performed on male CD-1 mice and compared to two groups: male mice undergoing sham surgery and male mice undergoing partial bladder outlet obstruction with castration. We measured serum testosterone levels in castrated mice and found that levels dropped to (<20 pg/dL) within 24 h after orchiectomy. The effects of obstruction on bladder function was determined utilizing urodynamic studies in these three groups. Figure 1A demonstrates elevated peak voiding pressures 1 week post-obstruction in the PO and CPO mice as compared to sham surgery mice. Representative urodynamic tracings also demonstrate instability at 1 week in PO and CPO mice (Figure 1C). VSOP performed at 1 week following obstruction reflect the effect of obstruction on PO and CPO mice that is not seen in sham surgery mice (Figures 1B,D). Thus, our model of PO had significant functional effects on micturition in both PO and CPO mice 1 week following surgery.

Though obstruction in CPO mice was demonstrated at 1 week, the effects of obstruction were resolved by 4 weeks. VSOP done at 4 weeks post-obstruction demonstrate that PO mice have persistent obstructive voiding patterns with smaller maximum voided volumes than CPO mice. At the same time point, CPO mice recover maximum voided volumes similar to sham surgery mice (Figures 3A–C). The overall pattern on VSOP of CPO mice have returned to normal while PO mice continue to demonstrate indiscreet spraying of urine indicating loss of bladder control.

### Bladder Remodeling following PO Is altered by Castration

**Figure 2:**
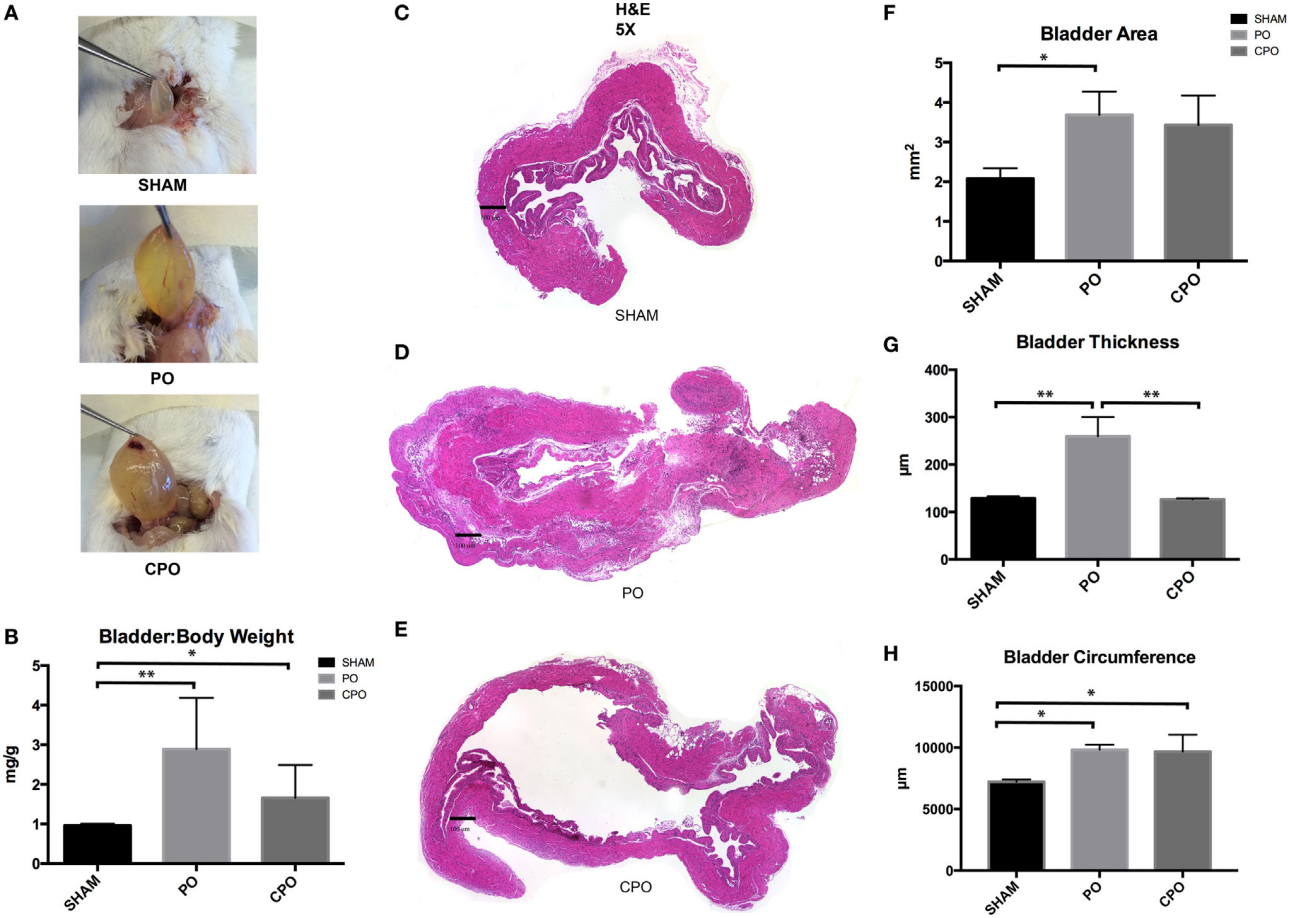
Bladder anatomy and wall thickness after PO at 1 week. **(A)** Photographs of bladders as they are excised from mice. **(B)** Bar graph showing bladder weight (mg) to body weight (g) in Sham, PO, and CPO mice. **(C–E)** Images of H&E stained sections of bladders from Sham **(C)**, PO **(D)**, and CPO **(E)** mice. **(F)** Bar graph showing mean total area of sections from Sham, PO, and CPO bladders. **(G)** Bar graph showing mean bladder wall thickness in Sham, PO, and CPO bladders. **(H)** Bar graph showing mean circumference of bladder sections from Sham, PO, and CPO mice. Error bars are SEM. Statistical significance is indicated above bars with an asterisk (*P < 0.05, **P < 0.01).

We found that effects on bladder function were correlated with macroscopic changes in the mouse bladder on PO and CPO mice. At the time of mouse sacrifice at 4 weeks following PO, sham bladders appeared normal in size, but PO and CPO bladder were grossly enlarged (Figures 2A,C–E). Following dissection of the bladder distal to the bladder neck ligature, bladders were expressed of urine and weighed. Here, we found increased bladder weight in the PO mice compared to both the sham surgery and CPO mice. CPO mice did not demonstrate increased weight in comparison to sham surgery mice (Figure 2B). Histologic evaluation of bladders of these mice was performed with H&E staining and bladder area, circumference, and thickness were compared. Both CPO and PO mice demonstrated increased area and circumference compared to sham surgery mice, reflecting changes from elevated bladder pressures following PO (Figures 2F,H). However, increased bladder thickness was only seen in PO mice (Figure 2G). This correlated with the lower bladder weights in CPO mice, suggesting that the increase in bladder size is primarily due to “stretching” of the bladder muscle as opposed to true bladder muscle hypertrophy or hyperplasia.

**Figure 3:**
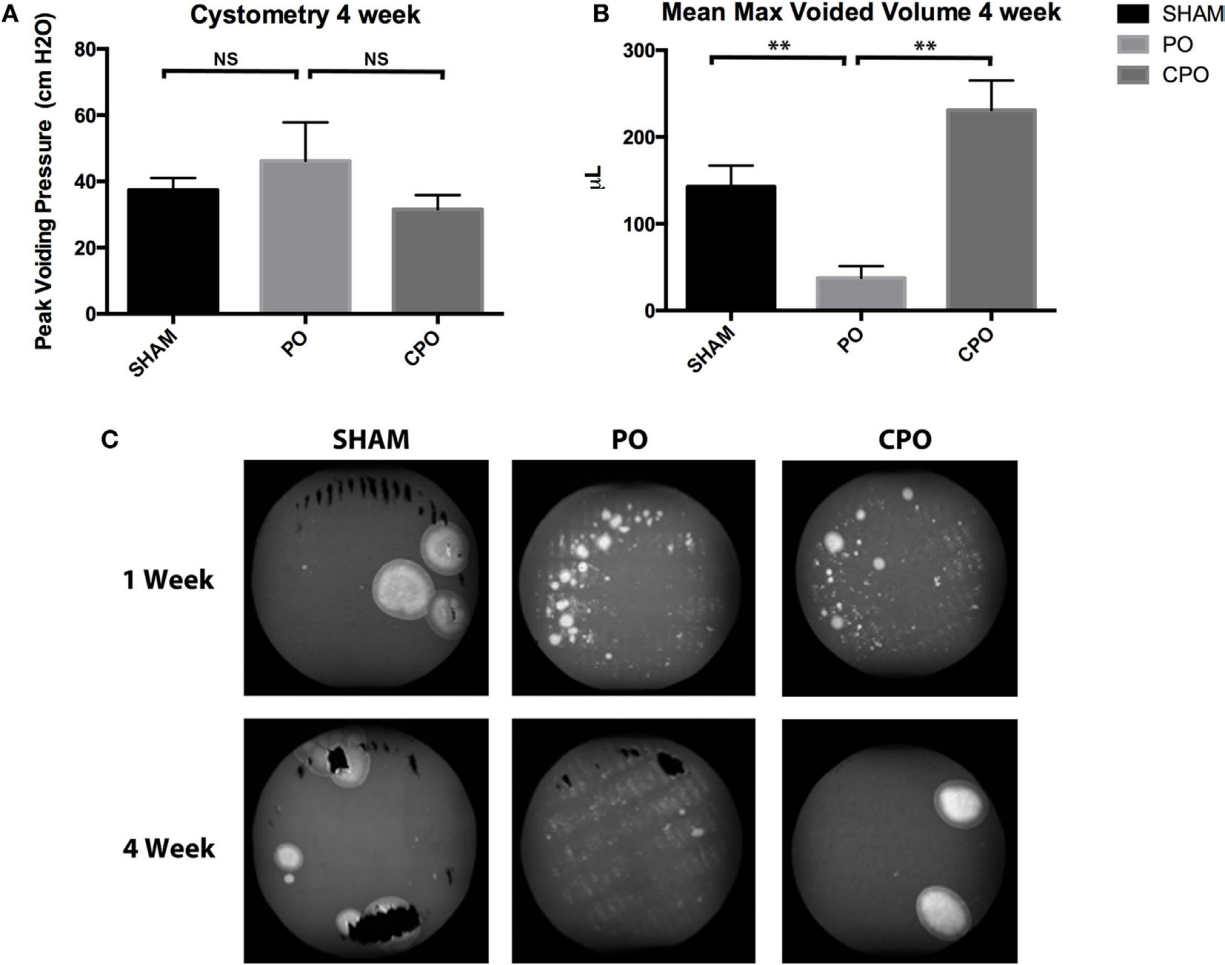
Cystometry **(A)** and mean max voided volumes **(B)** 4 weeks after Sham or PO. **(C)** Representative images of voiding stains from Sham, PO, or CPO mice 1 and 4 weeks after surgery. Error bars are SEM. Statistical significance is indicated above bars with asterisks (**P < 0.01). NS indicates that the difference was not statistically significant.

### Bladder Fibrosis Increases following Partial Bladder Outlet Obstruction

**Figure 4:**
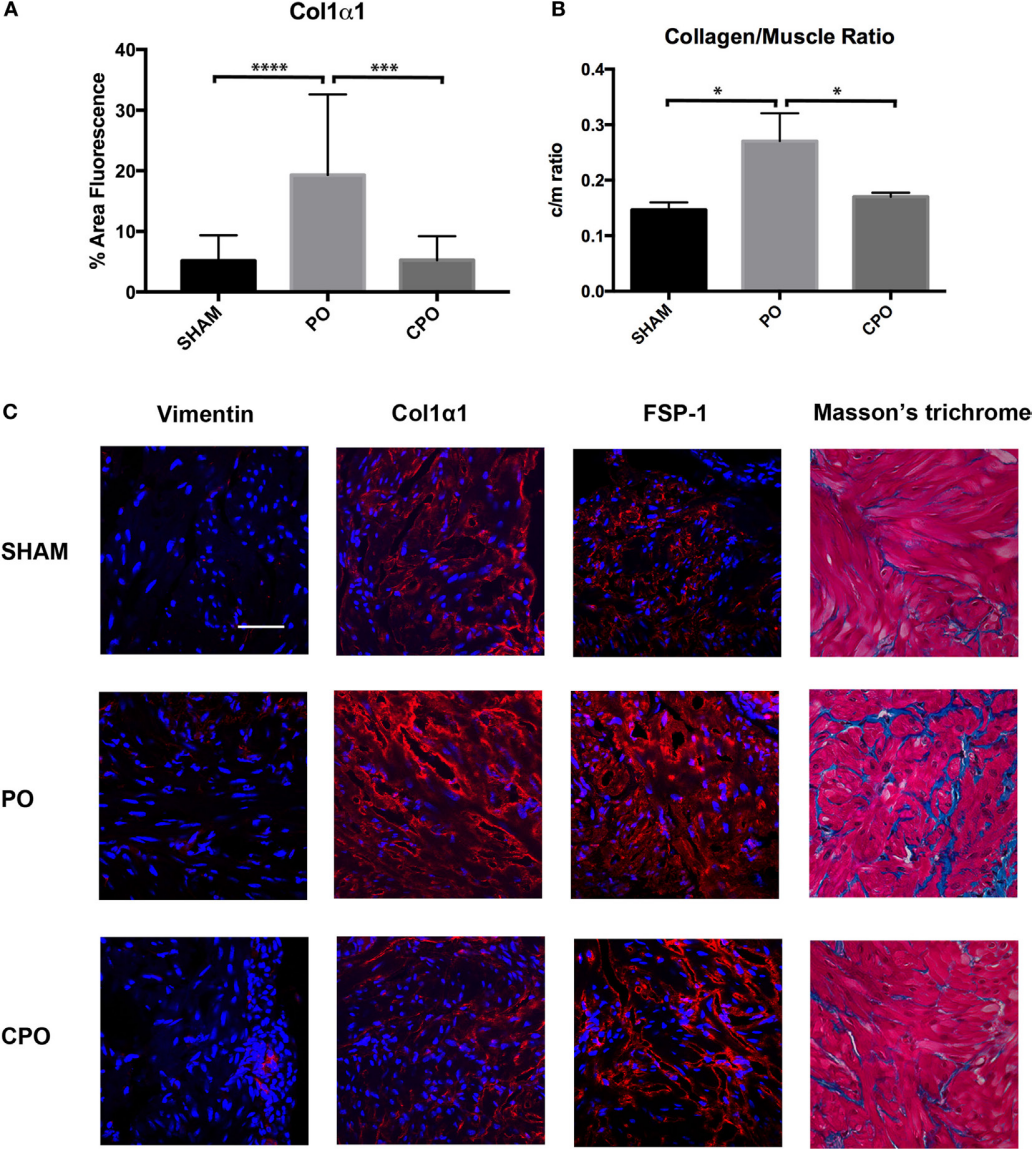
Presence of fibrotic proteins in the bladder wall after PO. **(A)** Bar graph showing mean percent area fluorescence for Col1α1. Significance is indicated by ***P < 0.001 or ****P < 0.0001. **(B)** Bar graph showing Col/SM ratio in Sham, PO, and CPO bladders. Significance is indicated by *P < 0.05. **(C)** Representative immunofluorescence images for Vimentin, Col1α1, FSP-1, and Masson’s trichrome. Antibody stain is red and DAPI counterstain is blue. Rows of panels are sections from (top to bottom) Sham, PO, and CPO. Rows are indicated on the left. Magnification bar in top left panel is 100 µm and represents the magnification for all panels.

In order to investigate if the increase in bladder mass and bladder wall thickness was due to solely muscular hypertrophy/hyperplasia or if a component of fibrosis was involved, histologic examination of sham, PO, and CPO mouse bladders was performed at 4 weeks following obstruction. This was done utilizing both Masson trichrome staining as well as fibrosis marker-specific immunofluorescence (Figure 4). As expected, in PO mice, following obstruction, the bladders were found to have increased Col/SM ratios. With castration, the increased Col/SM ratio was no longer found (Figure 4B). Though the luminal bladder volume was increased in CPO mice, the muscular layer did not demonstrate hypertrophy as evidenced by having the same bladder wall thickness as sham surgery mice. Furthermore, evaluation of fibrosis specific markers Vimentin, Col1α1, and FSP-1 by immunofluorescence demonstrated increased expression in PO mice compared to sham mice, with a corresponding decrease in expression in CPO mice. It appears that castration creates conditions in which non-fibrotic bladder remodeling occurs, arguing for a potential role of testosterone specifically in mediating a fibrotic response to bladder injury.

### Introduction of Exogenous Testosterone Reverses the Beneficial Effects of Castration following Partial Bladder Outlet Obstruction

**Figure 5:**
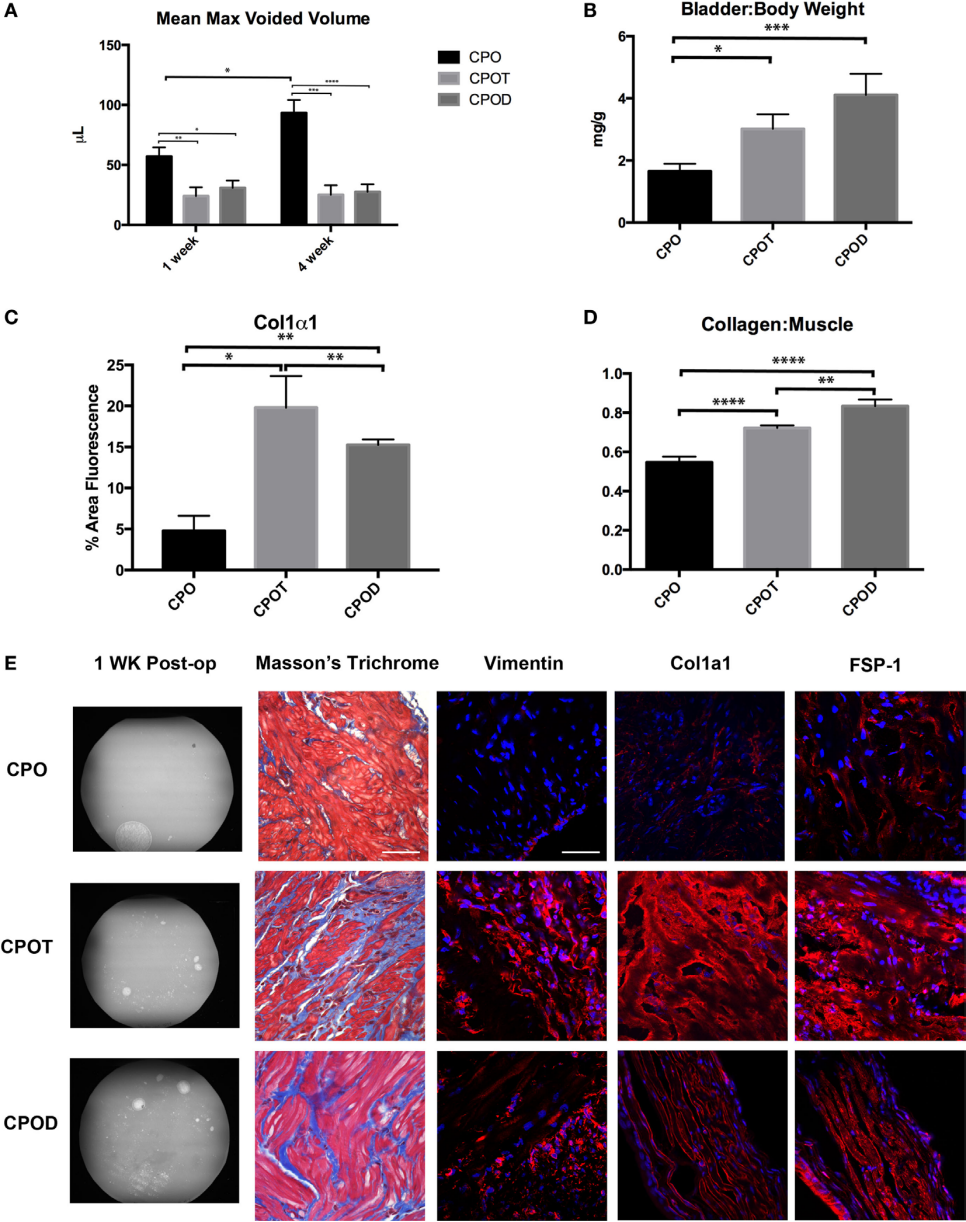
Effects of supplementing testosterone in castrated mice. Bar graphs showing mean max voided volumes **(A)**, bladder to body weight ratio **(B)**, immunostaining for Col1α1 **(C)**, and Col/SM ratio **(D)**. **(E)** Representative images of VSOP, Masson’s trichrome, Vimentin, Col1α1, and FSP-1 staining in CPO, CPOT, and CPOD mice. Statistical significance is indicated by *P < 0.05, **P < 0.01, ***P < 0.001, or ****P < 0.0001.

We have shown that castration in the setting of PO will attenuate the post-obstructive remodeling in regard to bladder hypertrophy/hyperplasia and fibrosis. To demonstrate that this is a testosterone-specific response, following castration, a cohort of mice underwent PO and testosterone supplementation. Here, we used either testosterone or its reduced product dihydrotestosterone (DHT). DHT has 10 times the affinity for the androgen receptor compared to testosterone and cannot be aromatized to estrogen, reducing the likelihood that the changes that occur from testosterone supplementation are due to increasing estrogen levels. Max voided volumes as determined by VSOP were significantly lower in CPO plus testosterone (CPOT) or CPO plus DHT (CPOD) mice as compared with CPO mice (Figure 5A). Macroscopic and microscopic changes seen in PO mice (but not CPO mice) reappeared in CPOT and CPOD bladders. Bladder weights were significantly increased in CPOT and CPOD mice as compared to CPO mice (Figure 5B). CPOT and CPOD bladders also had increased Col/SM ratios compared to CPO (Figure 5D). Fibrosis markers were also increased in CPOT and CPOD as compared to CPO bladders as was reaction to Masson’s trichrome stain (Figures 5C,E). These findings demonstrate that exogenous replacement of testosterone in castrated male mice fully restored the pathologic effects of obstruction to the urinary bladder. In sum, the effects of castration and testosterone replacement strongly support a role for male androgen in the pathogenesis of bladder obstruction.

## Discussion

Boys born with PUV undergo surgical resection of the obstructive leaflets soon after delivery. However, despite alleviating the bladder outlet obstruction in these children, many patients with PUV progress to bladder deterioration requiring clean intermittent catheterization to adequately empty the bladder (3). These changes were often seen following puberty. The clinical changes in patients with resected valves were previously attributed to physiologic changes associated with adolescence such as increased urinary filtration and not to hormonal changes (1, 5, 9). However, our findings suggest that androgen may play a more important role than previously appreciated. We find that the presence or absence of androgen specifically affects the cellular changes that occur after obstruction, in particular fibrosis.

Information from studies in which testosterone was supplemented in patients with lower urinary tract disease might give insight on the effects of androgen on bladder disease. These data are relatively sparse, coming largely from retrospective or prospective observational studies and studies in which testosterone was administered to hypogonadal or eugonadal men. However, these studies suggest that testosterone supplementation does not worsen LUTS (10, 11). Specifically, multiple studies have demonstrated an improvement in IPSS score in men receiving androgen supplementation, though many of these did not specifically study men with known bladder outlet obstruction, but rather include a cohort treated with testosterone supplementation and having varying degrees of LUTS at baseline. One study excluded those patients with known bladder neck obstruction. Koritsiadis et al. did specifically compare symptoms and urodynamic parameters on pressure-flow studies of hypogonadal and eugonadal men and found serum testosterone level to be negatively correlated with detrusor pressure at max flow, but not IPSS or any other urodynamic parameters (12).

The effect of testosterone on bladder detrusor muscle has also been studied in animal models. Tek and colleagues found that testosterone supplementation of surgically castrated rats resulted in a decreased collagen to smooth muscle ratio and increased cystometric capacity (13). Shortliffe et al. (14) found that testosterone replacement in surgically castrated rats was also associated with increased bladder smooth muscle as well as increased bladder mass (14). Together these studies indicate that in unobstructed rats, testosterone stimulates detrusor smooth muscle growth through a still unclear mechanism. In an ischemia-reperfusion bladder injury model in male rabbits, Chuang et al. demonstrated that castration was protective by decreasing bladder fibrosis and decreased expression of various fibrosis-related markers (8). Testosterone supplementation of these castrated rabbits led to increased detrusor fibrosis following bladder injury. Our findings mirror these findings and represent the first study of effect of testosterone on the response to bladder injury from PO in juvenile mice.

At the same time, the paucity of findings in the literature made us surprised by our initial findings. One potential concern we had was that the mice were not sufficiently obstructed and that our findings in castrated mice represented a group in which obstruction simply did not occur. In the end, we know this is not true because bladders in the CPO group initially had voiding stains like the obstructed group, had abnormal pressures by cystometry, and had remodeled bladders. In fact, the bladders from CPO mice were very different than shams, indicating that the PO surgery produced profound effects. Our interpretation of these findings is that in the hours following the surgery, pressure increases within the bladder wall and that, in normal mice, collagens and other stiffening matrices are constructed to prevent rupturing of the bladder into the abdomen. Bladders from CPO mice had very little deposition of collagen within the detrusor muscle but at the same time, became very large with thin walls. It is possible that when the fibrotic response is impaired in a high pressure bladder, it stretches to accommodate the urine that accumulates. This is typical of what we observed in CPO, but not PO and CPOT bladders. Thus, we hypothesize that the role of testosterone may specifically be in mediating the fibrosis in the bladder wall.

While our work specifically utilized a bladder model to determine the role of testosterone on muscle fibrosis in response to smooth muscle injury, we believe that this research will prove to be important in many fibrotic disease processes that have a sex association. In fact, this has been supported by a few studies in the literature. Cho et al. demonstrated that orchiectomy attenuated renal fibrosis seen after ureteral obstruction. In a mouse model of unilateral ureteral obstruction, kidneys from male mice demonstrated increased expression of alpha-SMA and collagen deposition (15). Similar to our bladder model of obstruction, this fibrotic response was minimized if concomitant orchiectomy was performed with ureteral obstruction. Analogous findings were reported in a mouse model of cardiac fibrosis secondary to pressure overload. Here, fibrotic changes in the left ventricle were attenuated by orchiectomy. This included a blunting of the TGFβ signaling pathway, known to be important for promoting myofibroblast differentiation (16). Age-related cardiac and renal fibrosis was also attenuated by orchiectomy in male mice (17). Furthermore, in a study by Zhu and colleagues, the androgen receptor on vascular smooth muscle cells was implicated in testosterone-stimulated smooth muscle calcification. Using a vascular smooth muscle cells derived from a smooth muscle-specific androgen receptor knockout mouse, calcification was blunted in response to testosterone treatment compared to wild type smooth muscle cells (18). Thus, an increasing number of studies, including ours, are finding an association between testosterone and the pathological changes associated with fibrosis.

The mechanism behind the role of testosterone in modulating the detrusor response to injury remains unexplained. Further work investigating the downstream effects of testosterone in detrusor muscle could reveal novel therapeutic targets that could be utilized to prevent or at least ameliorate the adverse tissue remodeling seen in the setting of PO. Additionally, dose–response studies could be conducted to determine at what serum concentrations of testosterone lead to adverse remodeling. Estrogen has been shown to attenuate tissue hypoxia and associated detrusor injury in animal models. Further study of the interplay between testosterone and estrogen in modulating the detrusor muscle response to injury may also help to further elucidate this pathway.

## Conclusion

Juvenile male mice with partial bladder outlet obstruction have smaller high pressure bladders that are fibrotic. Castration was associated with larger voided volumes, lower bladder weight, and less detrusor fibrosis. Replacement of testosterone in castrate mice reversed these effects. This indicates that testosterone plays a role in modifying alterations to detrusor muscle after partial bladder outlet obstruction in mice. Thus, we suggest that increased levels of testosterone in boys with PUV may contribute to the decreased bladder function.

## Ethics Statement

This study was carried out in accordance with the recommendations of surgical guidelines, Animal Care and Use Committee. The protocol was approved by the Animal Care and Use Committee.

## Author Contributions

AF, PF, DB, NK, and GS data collection and analysis. AF, PF, RD, and EG conception, design, and manuscript writing.

## Conflict of Interest Statement

The authors declare that the research was conducted in the absence of any commercial or financial relationships that could be construed as a potential conflict of interest.
